# Clinical and Epidemiological Approach to Delirium in an Acute Care Unit: A Cross-Sectional Study

**DOI:** 10.3390/ijerph19159406

**Published:** 2022-07-31

**Authors:** Pablo Jorge-Samitier, Raúl Juárez-Vela, Iván Santolalla-Arnedo, Ana Cobos-Rincón, José Ángel Santos-Sánchez, Vicente Gea-Caballero, Pedro José Satústegui-Dorda, Ana Anguas-Gracia, Clara Isabel Tejada-Garrido, Fernando Urcola-Pardo, María Teresa Fernández-Rodrigo

**Affiliations:** 1 Hospital Clínico Lozano Blesa, Avda. San Juan Bosco, 15, 50009 Zaragoza, Spain; pjorge@salud.aragon.es; 2 Department of Physiatry and Nursing, University of Zaragoza, 50009 Zaragoza, Spain; 3 GRUPAC, Department in Nursing, University of La Rioja, C/Duquesa de la Victoria 88, 26004 Logroño, Spain; raul.juarez@unirioja.es (R.J.-V.); ivan.santolalla@unirioja.es (I.S.-A.); clara-isabel.tejada@unirioja.es (C.I.T.-G.); 4 Faculty of Medicine, University of Salamanca, 37008 Salamanca, Spain; 5 Radiodiagnostic Unit, Castilla Leon Health Care Service (SACYL), University Hospital of Salamanca, 37008 Salamanca, Spain; 6 Community Health and Care Research Group, Faculty of Health Sciences, Valencia International University, 46003 Valencia, Spain; vagea@universidadviu.com; 7 Research Group Water and Environmental Health (B43_20R), Department of Physiatry and Nursing, University of Zaragoza, Calle Domingo Miral, s/n, 50009 Zaragoza, Spain; pjsd@unizar.es (P.J.S.-D.); aanguas@unizar.es (A.A.-G.); furcola@unizar.es (F.U.-P.); maitefer@unizar.es (M.T.F.-R.)

**Keywords:** delirium, aged, confusion, mental health, hospitalization

## Abstract

During hospital admissions, the union of various factors, those related to acute pathology, dependency conditions, cognitive impairment, change of habitual environment, and others, can cause delirium. Acute delirium in the elderly (ADE) occurs in around a third of patients over 70 years of age. The syndrome generates serious complications that increase hospital morbidity and mortality and a high cost for the health administration. This study aimed to determine the clinical and epidemiological profile of ADE in an internal medicine unit. A descriptive cross-sectional study was carried out using a convenience test. A total of 356 patients participated between September and November 2021. Sociodemographic variables, predisposing and precipitating factors of ADE, methods of action against ADE, and the impact on functional and cognitive deterioration were analyzed. A total of 35.1% of the patients developed ADE, mostly of the hyperactive type and of nocturnal appearance. ADE was mainly treated with psychoactive drugs and 22% required mechanical restraint, with non-pharmacological preventive strategies, support, and caregiver training being the main tools for controlling ADE during hospital admission.

## 1. Introduction

During hospital admissions, the union of several factors such as those related to acute pathology, dependency conditions, cognitive impairment, the environment, and other associated factors can cause presentations of temporospatial disorientation and psychomotor depression and in some cases, even hypoactivity. This clinical syndrome is what is known as “Acute Delirium in the Elderly” (ADE) or “Acute Confusional Syndrome” (ACS). According to the criteria of the Diagnostic and Statistical Manual of Mental Disorders V (DSM-V), it is a neurocognitive disorder characterized by the presence of four key characteristics: altered level of consciousness, the presence of cognitive and perceptive alterations, and sudden onset and fluctuating character [1]. Regarding its etiology, it is multifactorial, resulting from the interaction of a prior vulnerable neurobiological state and one or multiple triggering or precipitating agents such as an acute illness or prescribed medication. Clinically, sleep–wake cycle disorders (daytime sleepiness and nighttime insomnia), short-term memory disorders, and incoherent and disorganized thinking are characteristic, frequently appearing alterations in perception and hallucinations [2,3,4,5]. ADE can be projected in hyperactive (agitation and hallucinations), hypoactive (psychomotor slowdown, apathy, lethargy), or mixed form, with the hypoactive type being an underdiagnosed condition [2,4,5].

ADE is a frequent and harmful complication among the elderly and is also a psychogeriatric symptom of a medical emergency [4,6,7,8,9,10,11,12]. We can affirm that it is one of the most important complications during hospitalization of the elderly population, with an incidence that varies between 15–64% [13,14]. It is the second most prevalent psychiatric syndrome after depressive disorders in the hospital setting and its incidence increases with age, ranging between 6% and 56% in patients over 65 years of age during the hospitalization period [6,12,13,14,15,16]. Its presence in the elderly is associated with high morbidity and higher mortality (between 9–34%) [2,3,9,17]. It is also a predictor of institutionalization and rehospitalization and implies an increase in the days of hospital stay and an increase in associated health costs [9,12,14,18].

For the health system, it is an important public health problem, given its relevant negative impact at the bio-psycho-social level on the integrity of the patient and their environment, it is also a factor of intense stress both in the personnel who care for them and in the patients, family, and informal caregivers who witness it [7,19,20]. Despite this, health personnel often do not give it due importance, leaving this syndrome undiagnosed. There is a false belief that this situation is inherent to the characteristics of the patient and cannot be avoided [21]. Studies on ACS report that there are a series of predisposing factors and precipitating factors that should be taken into account during admissions in order to prevent and/or treat ACS. Preventive intervention contributes to improving the development, prognosis, and evolution of hospital admissions. Around 30–40% of ACS episodes can be prevented by non-pharmacological measures. Measures such as the identification of the risk of suffering from ADE, environmental measures, temporal-spatial orientation, ensuring night rest, medication review, adequate hydration, cognitive stimulation, ensuring correct vision and hearing, and early mobilization [4,6,7,10,11,14,20,21,22]. The objective of this study was to determine the epidemiological and clinical profile of ADE in an internal medicine unit and to analyze the associated factors.

## 2. Materials and Methods

### 2.1. Study Design and Setting

We performed an observational, descriptive, and cross-sectional study. Data collection was carried out between 1 September 2021 and 31 November 2021.

### 2.2. Data Collection

A non-probabilistic, convenience, consecutive sampling was carried out among all those admitted in that period at the Internal Medicine Unit B of the Hospital Clínico Universitario Lozano Blesa in Zaragoza (Spain) with the following inclusion criteria: patients with age > 65 years, with at least 3 days of evolution of hospital admission and who had signed the informed consent. The exclusion criteria were as follows: patients admitted in extremis and in a near-death state. Patients who met the inclusion criteria were assessed using a self-prepared questionnaire previously used and divided into three blocks [23]. The first block dealt with sociodemographic variables (age, sex, place of residence, people living together) and the clinical and dependency conditions before hospital admission related to predisposing factors for ADE. The presence of multiple pathologies was analyzed according to the classification of the Andalusian Health Service [24]. Functional assessment was performed using the Barthel scale [25]. The assessment of cognitive impairment was conducted using the Pfeiffer scale [26]. The assessment of the state of hydration and nutrition was performed using the modified Norton scale of INSALUD [27].

The second block was designed to be completed if a possible ADE was detected during hospital admission. For this, the Confusion Assessment Method (CAM) scale had to be completed to confirm the diagnosis. The CAM scale is recognized as the best tool for the detection of ADE with a sensitivity of 93% and a specificity of 89% [28]. It is easy to administer [29] and has become the most widely used ADE detection instrument [30,31]. Then, in the last block, the variables related to the possible precipitating factors that could have triggered the condition (presence of serum therapy, oxygen therapy, bladder catheterization, raised railings, presence of a diaper, pain or discomfort, dyspnea, dehydration, metabolic alteration) were completed. In addition, in this part of the questionnaire, the action taken during the delirium process had to be completed: verbal and pharmacological restraint and, if necessary, mechanical restraint [5,32]. The questionnaires were completed by nurses specifically trained for it.

### 2.3. Statistical Analyses

Statistical analysis was performed using the IBM SPSS Statistics 25.0 program (New York, NY, USA). For all analyses, if the *p*-value was less than 0.05, it was considered statistically significant. Descriptive statistics such as the frequency measures (mean and standard deviation) and percentages were used to describe the appearance of the different sociodemographic and clinical variables related to ADE. In addition, in order to evaluate the existence of a significant relationship between the different variables and the appearance or not of ADE, or how these variables affect the development of ADE, an analysis was performed using the Chi-square test. In cases where the number of cases was less than 5, and the Chi-square test was not reliable, Fisher’s exact test was chosen.

### 2.4. Ethical Consideration

All respondents or their families were informed about the objectives and requirements of the study, assuring them of the confidentiality of the data according to Organic Law 3/2018, of December 5, signing the informed consent established in the protocol approved by the Committee of Ethics and Research of Aragon (CEICA) no. 115/2016.

## 3. Results

Table 1 shows the sociodemographic description of the 356 cases, among whom the male sex predominated, with a mean age of 83.61 years (+7.56), with 46.1% being > 86 years.

Regarding the reasons for admission, heart failure (HF) stands out, followed in second place by admissions due to respiratory processes, and then processes that entered the study (anemia, constitutional syndrome, pain), and finally, infections of the urinary system with no statistically significant relationship being established between the admission diagnosis and the development of ADE, although high rates of ADE have been established in several diagnoses such as HF, urinary disorders, respiratory problems, pain, dehydration, or fever. Table 2 shows the relationship between the predisposing factors in the appearance of ADE, highlighting 82.9% of patients with multiple pathologies, 30.3% with moderate dependence, and 47.3% with the use of hypnotics, in chronic form.

Among the patients who suffered from ADE, 91.2% were over 75 years old and were mostly male and had multiple pathologies. About 70% had moderate or severe functional dependence, moderate, or severe cognitive impairment, and almost half had a history of ADE. All of this is represented in Table 2.

Of the total number of patients, 35.1% presented ADE, of which more than half were classified as hyperactive ADE and started at night. A total of 49.6% of the cases occurred on the same day of hospital admission. During the stay, 66.4% of the cases occurred between the first 2 days and almost 80% between the first 3 days, the figures dropping notably from the fourth day. Table 3 presents this data together with the precipitating factors that could trigger the ADE presentation.

Only 22.2% of the patients required pharmacological support on the day of the ADE event, while the rest required pharmacological support for more days, even during the entire hospital stay. The drugs of choice to treat them were haloperidol and quetiapine. The ADE presentations were repeated for at least 2 days in a row in 35.7% of the cases, for 3 days in 21.4%, and 4 days in 14.3%. After the administration of medication, during the resolution phase of the ADE presentation and psychomotor agitation, 42.6% went into a situation of hypoactivity. The ADE event was repeated at least two times in 32.7%, three times in 21.8%, four times in 14.5%, and between 5 and 16 days in 27.1%. In the cases in which mechanical restraint was necessary, it lasted between 1 and 3 days in 45.9% and the rest between 4 and 10 days in a row. The use of pharmacological restraint was necessary without differences in the baseline cognitive level, while the use of mechanical restraint was more likely as cognitive impairment increased. Table 4 shows the possible relationship between the type of care exercised before the appearance of ADE and the degree of functional and cognitive impairment, age, sex, and whether it was accompanied.

Table 5 shows the impact of the appearance of ADE on the length of hospital stay, the impact of early mobilization, and the level of functional dependence.

## 4. Discussion

The objective of this study was to analyze the predisposing factors with which patients were admitted to our internal medicine unit, especially to facilitate the delimitation of the most vulnerable population to suffer from ADE, to identify what precipitating factors are more present in our unit and what is the method of approaching ADE by health personnel to facilitate care aimed at preventing ADE and improving the quality of care offered. The main results observed are in line with the evidence analyzed and raise a real possibility of improvement in the prevention of delirium through the training of professionals and with the indispensable collaboration of family and informal caregivers. The percentage of DAA above 30% could be directly related to the increase in the average age and high percentages of dependency and cognitive impairment at admission. Comparing it with a previous study carried out in the same unit with a population diagnosed with COVID-19, with a lower mean age (77 years) and less functional dependence and cognitive impairment, we observed slightly lower DAA results of 29.6% [23]. These results could be compared with those of Vazquez et al. [9], who presented the results of a delirium incidence of 43%, although various studies such as the review by Fernandez-Moreno et al. [13] and the works by Inouye et al. [33] expanded the range between 15 and 65%. In our study, a significant relationship appeared between age, level of dependency, and cognitive status with the appearance of ADE. Male sex, the presence of potentially disabling multiple pathologies, having suffered from ADE on previous occasions, and chronic daily use of hypnotics also had a strong relationship. All of these factors would be in line with the published evidence, highlighting the percentages of the chronic use of hypnotics, both in the general population at admission with 57% and those who subsequently developed ADE who consumed them daily with 68% [7,10,13,34,35,36]. Comparing these data with those of our previous studies carried out in the same unit that applied the drugs in 32.4% of patients hospitalized for COVID-19 [23], which rose up to 40% among those who developed ADE and 35% relating to HF and sleep quality disorder [37], a significant increase in the use of these drugs was perceived, whose differences could be found in age, functional, and cognitive impairment.

On the other hand, our study did not show a relationship between nutritional status, hydration, or sensory impairment (*p* = 0.59), perhaps because the cases of malnutrition and dehydration diagnosed on admission were few since the unit is already including the practice of fitting hearing aids and glasses to patients who used them regularly.

Regarding the precipitating factors of ADE, we only found a relationship of statistical weight between the use of serum therapy, the placement of railings as an element of individual protection, and the use of underpads to control excreta, all of them modifiable factors, although conditioned by the disease. This appears to be a factor with less statistical power, but with striking figures, the withdrawal of hypnotic medication at hospital admission (*p* = 0.06), and not establishing a relationship with other factors that appear as important in the literature such as fever, pain, or metabolic alteration [6,7,10,31,34,35,36]. This deviation could be caused by detection errors as there is no monitoring of the analytical parameters.

Regarding the description of the characteristics of the moment of appearance of ADE, our study agrees with the published evidence [8,13,17,35]. Its appearance was more frequent at night and within the first 48 h. For all of these reasons, those first days are key to its possible prevention. In addition, our study is consistent with the results of previous evidence in relation to follow-up, establishing it as a protective and fundamental factor in the prevention and detection of ADE. Hyperactive ADE was also more frequent in our study, but the previous training of the nursing staff who had to diagnose the condition allowed for the classification between hyperactive, hypoactive, and mixed, minimizing the traditional underdiagnosis of the hypoactive and mixed subtypes [4,8,31,38].

The drugs chosen to control ADE are appropriate according to the clinical practice guidelines, mainly parenteral haloperidol and oral quetiapine. The guidelines on the use of haloperidol and antipsychotics mention the ability to generate sedation as a side effect, among others. There could be a relationship between the administration of medication to control hyperactive ADE and subsequent hypoactivity, causing the mixed and alternate types of hyperactivity and hypoactivity, although in our case, we did not find a statistically significant relationship (*p* = 0.09). Although there may be some conflict in the duration of therapy in the following days, its use is recommended for the shortest possible time and at the lowest possible dose [5,16,21,35,39,40,41].

In addition to mechanical restraint, this was considered pertinent in 23% of cases and could last up to 19 days in a row, although in 45%, it was present for one, two, or three days in a row at most. There is enormous variability in its use in long-term institutions, based on studies carried out in different countries, from 6% in Switzerland to 31% in Canada, reflected in the guide to alternatives to the use of restraints of the Association of nurses in nursing, Ontario [42]. In our study, conducted in a COVID-19 population with a lower mean age and with better dependency conditions, the use of mechanical restraint was only necessary in 9% of cases (23), while the use of psychoactive drugs remained similar around 60%, although the clinical practice guidelines require a limitation of its use in time and form since it is considered as an element contrary to the dignity, the self-esteem of the person, and the right to autonomy, in addition to increasing the risk of suffering from other accidents such as lacerations, strangulation, pain, and agitation [42,43,44,45,46].

The clinical guidelines that address the possibilities in the management of ADE do not establish a typical profile or characteristics of people who can predominantly see their delirium resolved with non-pharmacological, pharmacological, and/or mechanical restraint measures [28,39,41,42]. In this sense, in our study, there did not seem to be a relationship between age, sex, or level of dependency and the restraint method used to control ADE (*p* > 0.05), except for cognitive impairment (*p* = 0.01), which seems to have been chosen as a factor that decisively influences the pharmacological intervention, and/or accompanied by mechanical restraint.

Our study defends the relationship between the appearance of ADE and the lengthening of the hospital stay and the difficulty in carrying out early mobilization could be one of the causes, an issue endorsed by Rubin et al. [10] in the works aimed at the development of the Hospital Elder program Life Program (HELP).

On the other hand, according to different studies on the viability of the HELP(10) program and those analyzed in the review by Fernández Moreno [13] or that of Aguilar et al. [28], between 30 and 40% of ADE cases could be avoided with preventive measures. Another of the general recommendations in the care of geriatric patients aimed at preventing functional and cognitive deterioration and the appearance of ADE itself, is early mobilization endorsed by the clinical practice guidelines on the prevention, diagnosis, and treatment of delirium of the National Institute for Health and Clinical Care Excellence [11]. The results of our study maintain this line, establishing a significant relationship between early mobilization and the non-appearance of ADE, behaving as a preventive factor. In this sense, although in our previous study that related ADE and COVID-19 [23] where the main problem was the lack of follow-up of the patient since early mobilization was conditioned by the presence and collaboration of relatives or caregivers, in the current study, in which the accompaniment was present, the fundamental problem was the training of these same caregivers and the organization of the unit itself and the lack of personnel that prevented the necessary mobilizations for the correct clinical evolution.

The non-probabilistic, convenience sampling procedure can limit the statistical effects of the results. Due to the characteristics of the epidemiological design, we cannot establish cause–effect relationships, although we hypothesize that one of the issues highlighted by the evidence on ADE is that it is closely related to institutionalization and care overload in the family.

## 5. Limitation

We have to take into account that due to the design of the study, we could not establish cause–effect relationships. However, we want to emphasize that a large number of patients admitted with heart failure with a history of disease decompensation may be a predisposing factor in greater measure than other pathologies for the appearance of ADE.

## 6. Conclusions

Age, functional, and cognitive impairment seem to behave as the fundamental risk factors for suffering from ADE in an internal medicine unit. The prevention of ADE should focus on assessing the risk of suffering from ADE, on the establishment of organizational care measures that facilitate early mobilization, and temporospatial orientation, which limits physical restrictions and excess sensory stimulation, especially during sleep hours, in improving diagnostic capacity and promoting caregiver training in basic areas of ADE care. Based on the results of our study, we conclude that the administration of hypnotic drugs and mechanical restraints should be limited to the minimum necessary time.

## Figures and Tables

**Table 1:** The sociodemographic data of the total sample.

Characteristics	Variable	*n* (%) Mean ± SD
Age (interval)		83.61 ± 7.56
	65–75	68 (19.1)
	76–85	124 (34.8)
	>86	164 (46.1)
Sex	Male	187 (52.5)
	Female	169 (47.5)
Place of residence	Urban population	192 (54)
	Rural population	164 (46)
Co-residence	Spouse	133 (37.4)
	Son/Daughter/Other family	79 (22.2)
	Alone	28 (7.9)
	Caregiver	25 (7.9)
	Geriatric home	91 (25.6)
Medical background Pluripathology	<2 *	49 (13.7)
	x ≥ 2 *	295 (82.9)
**Reason hospital admission**	Heart failure	93 (26.1)
	Respiratory process	85 (23.9)
	Disease study	71 (19.9)
	Urinary diseases	57 (16)

* Number of potentially disabling pathologies. According to the classification of the Andalusian Health Service [24].

**Table 2:** The relationship of the predisposing factors between the total sample compared to the cohorts that presented with ADE and those that did not.

	*n* (%)	No DAA *n* (%)	DAA *n* (%)	Chi²/Fisher (*p*)
Age				
65–75	68 (19.1)	57 (24.7)	11 (8)	<0.001
76–85	124 (34.8)	87 (37.7)	37 (29.6)	<0.001
>86	164 (46.1)	87 (37.7)	77 (61.6)	<0.001
Male sex	187 (52.5)	112 (48.5)	75 (60)	0.038
Pluripathology	295 (82.9)	187 (81)	108 (86.4)	0.007
Severe dependence (Barthel < 35)	83 (23.3)	43 (18.6)	40 (32)	<0.001
Moderate dependence (Barthel 35–65)	108 (30.3)	54 (23.4)	54 (43.2)	<0.001
Mild dependence or independence	165 (46.4)	134 (58)	31 (24.8)	<0.001
Auditory sensory impairment	33 (9.3)	18 (7.8)	15 (12)	0.591
Visual sensory impairment	43 (12.1)	29 (12.6)	14 (11.2)	0.591
Auditory and visual sensory impairment	4 (1.1)	3 (1.3)	1 (0.8)	0.591
Medium nutritional status	166 (46.6)	111 (48.1)	55 (44)	0.357
Fair nutritional status	83 (23.3)	49 (21.2)	34 (27.2)	0.357
Dehydration	25 (7)	9 (3.9)	16 (12.8)	0.086
Daily use of hypnotics	204 (57.3)	119 (51.5)	85 (68)	0.009
Previous delirium episode	89 (25)	29 (12.6)	60 (48)	<0.001

**Table 3:** The characteristics of ADE and the presence of precipitating factors in the development of ADE.

	Total Sample (%)	No DAE (%)	DAE (%)	Chi²/Fisher (*p*)
Diagnosed ACS	108 (35.1)			
- Hyperactive			73 (58.4)	
- Hypoactive			16 (12.8)	
- Mixed			33 (28.8)	
- At night			71 (56.8)	
- On the admission day			62 (49.6)	
- Starting on the 2nd day of admission			21 (16.8)	
- Starting on the 3rd day of admission			16 (12.8)	
- Starting on the 4th day of admission or later			26 (19.8)	
Peripheral venous line	353 (99.2)	228 (99.6)	125 (100)	0.459
Saline solution	181 (50.8)	90 (39)	91 (72.8)	<0.001
Bladder catheterization	12 (11.1)	42 (18.3)	30 (24)	0.206
Oxygen therapy	185 (52)	115 (49.8)	70 (56)	0.281
Use of bed rails	285 (80.1)	167 (72.9)	118 (94.4)	<0.001
Use of diapers	285 (80.1)	170 (74.2)	115 (92)	<0.001
Wet diaper in patients with DAE	22 (6.2)	2 (33.3)	20 (19.6)	0.417
Fever	52 (14.8)	31 (13.7)	21 (16.8)	0.426
Pain	69 (19.7)	45 (20)	24 (19.2)	0.857
Metabolic disturbance	149 (45.8)	94 (44.6)	55 (48.3)	0.132
Daily use of hypnotics	204 (57.3)	119 (51.5)	85 (68)	0.008
Withdrawal of hypnotics on admission	53 (25.7)	24 (20)	29 (33.7)	0.064
Regular and poor nutritional status	88 (24.7)	51 (22.1)	37 (29.6)	0.372
Dehydration	25 (7)	3 (9)	16 (12.8)	0.003

**Table 4:** Influence of functional and cognitive impairment, age, and sex on the type of care provided to the DAE.

		Only Verbal *n* (%)	Verbal and Pharmacological *n* (%)	Pharmacological and Mechanical *n* (%)	(*p*)
Barthel Functional Assessment Index	Severe dependency	3 (17.6)	18 (28.6)	9 (39.1)	0.145
	Moderate dependency	8 (47.1)	33 (52.4)	12 (52.1)	
	Independence	6 (35.3)	12 (19)	2 (8.7)	
Age (intervals)	65–75	1 (5.9)	4 (6.3)	4 (17.4)	0.112
	76–85	3 (17.6)	19 (30.2)	10 (43.5)	
	>86	13 (76.5)	40 (63.5)	9 (39.1)	
Sex	male	12 (70.6)	33 (52.4)	18 (78.3)	0.065
	female	5 (29.4)	30 (47.6)	5 (21.7)	
Accompaniment	Only	8 (47.1)	15 (23.8)	12 (52.2)	0.065
	Family	7 (41.2)	25 (39.7)	5 (21.7)	
	Caregiver	2 (11.8)	22 (34.9)	5 (21.7)	
	Both	0 (0)	1 (1.6)	1 (4.3)	

**Table 5:** The evolution of hospital admission until discharge.

		Not DAE *n* (%)	DAE *n* (%)	(*p*)
Duration of admission	<7 days	91 (39.4)	34 (27.2)	0.038
	7–14 days	94 (40.7)	59 (47.2)	
	14–21 days	33 (14.3)	17 (13.6)	
	>21 days	13 (5.6)	15 (12)	
Early mobilization	1st week	163 (70.6)	50 (40)	<0.001
	2nd week	4 (1.7)	9 (7.2)	
	Impossibility	64 (27.7)	66 (52.8)	
Barthel (discharge)	Severe dependence (<35)	45 (20.8)	60 (53.5)	<0.001
	Moderate dependence (40–80)	80 (37.1)	45 (40.2)	
	Autonomy (>85)	90 (41.7)	7 (6.3)	

## Data Availability

Please contact the first author.
